# Malaria in infants aged less than six months - is it an area of unmet medical need?

**DOI:** 10.1186/1475-2875-11-400

**Published:** 2012-12-02

**Authors:** Umberto D’Alessandro, David Ubben, Kamal Hamed, Serign Jawo Ceesay, Joseph Okebe, Makie Taal, Eugene Kaman Lama, Moussa Keita, Lamine Koivogui, Alain Nahum, Kalifa Bojang, Aja Adam Jagne Sonko, Honorat Francis Lalya, Bernard Brabin

**Affiliations:** 1 Medical Research Council Unit, PO Box 273, Banjul, The Gambia; 2 Institute of Tropical Medicine, Nationalestraat 155, 2000, Antwerp, Belgium; 3 Medicines for Malaria Venture (MMV), PO Box 1826, 20 rte de Pré-Bois, 1215, Geneva 15, Switzerland; 4 Novartis Pharmaceuticals Corporation, One Health Plaza, East Hanover, NJ, 07936-01080, USA; 5 Ministry of Health and Social Welfare, The Quadrangle, Banjul, The Gambia; 6 Ministry of Health, BP 6623, Conakry, Guinea; 7 Centre de Recherches Entomologiques de Cotonou, Cotonou, Benin; 8 Centre National Hospitalier Universitaire Hubert K. Maga, Cotonou, Benin; 9 Child and Reproductive Health Group, Liverpool School of Tropical Medicine, Pembroke Place, Liverpool, L35QA, UK

**Keywords:** Malaria, Neonate, Congenital, Prevalence, Parasitaemia, Artemisinin-based combination therapy

## Abstract

Despite the protection provided by several factors, including maternal antibodies, the burden of malaria in young infants may be higher than previously thought. Infants with congenital or neonatal malaria may have a different clinical presentation than older children, and diagnosis may be confused with other neonatal diseases due to an overlap of clinical manifestations. In addition, there is little information on the use of artemisinin-based combination therapy in young infants. There is the need for a more accurate estimate of the parasite prevalence and the incidence of clinical malaria in infants under 6 months old, as well as a better characterization of risk factors, pharmacokinetic profiles, safety and efficacy of currently available anti-malarial treatments, in order to develop evidence-based treatment guidelines for this population.

## Background

Although the burden of *Plasmodium falciparum* malaria in children aged under five years is well documented [1], there is limited and contradictory information about the impact of the disease in infants under six months of age. Early studies concluded that malaria was uncommon in young infants and that clinical disease was of little importance [2-4], despite several early reports of a uniform rise in parasite prevalence during the first few months of life [5]. Young infants with malaria may have different clinical manifestations [6,7] and lower parasite densities [5] than older children. Nevertheless, even low-density infections (1–500 parasites/μL) in infants can result in anaemia if left untreated [8], and may rapidly progress to become life-threatening [9].

Neonatal malaria is considered a rare occurrence due to the protective effect of maternal immunity after birth. Maternal immunoglobulin G (IgG) antibodies are acquired by the fetus via the placenta *in utero*[10], although there is only limited evidence suggesting a protective role of these passively acquired antibodies against malaria parasites [11]. IgG levels are thought to decrease variably during the first year of life [12]. Neonates may also be protected through factors that inhibit parasite growth, such as lactoferrin (which binds iron) and secretory IgA, found in breast milk and in maternal and infant sera [13]. Parasite replication depends on the metabolic substrate para-aminobenzoic acid (pABA), which is present only in low levels in breast milk [10]. Haemoglobin F (HbF), present in high concentrations at birth [10], can inhibit parasite development [14] and can protect the infant in the first few months of life. The parasite antigen *P*. *falciparum* erythrocyte membrane protein-1 (PfEMP-1) mediates cytoadherence of infected red blood cells to the endothelial cells lining the blood vessels. HbF and maternal IgG act cooperatively to impair the cytoadherence of parasitized erythrocytes in the first few months of life by altering PfEMP-1 display on HbF RBCs, and by binding PfEMP-1 and preventing sequestration of parasitized RBCs, respectively [15]. HbF levels decline after a peak at 6 weeks of age [10], and as both HbF and IgG disappear from circulation, infant susceptibility to *P*. *falciparum* malaria increases [15].

Iron-deficiency anaemia is common in sub-Saharan Africa and can cause impaired cognitive and motor development, growth impairment and anorexia. Consequently, in many areas, iron supplementation is recommended in children below two years of age. However, iron status has been shown to have an effect on the risk of malaria in young infants, with iron deficiency potentially causing a decrease in malaria morbidity and mortality [16].

In Mozambique and Benin, placental malaria has been associated with shorter time to first episode of infant malaria [17,18]. The mechanisms underlying this association are unclear but may relate to prenatal foetal priming [19]. Therefore, some reduction of malaria risk in infants can probably be achieved by improving malaria control in late pregnancy.

## Burden of malaria in infants aged less than six months

Malaria in infants is classified according to the time of infection. Congenital malaria, defined as asexual parasites detected in the cord blood or in the peripheral blood during the first week of life [20], is due to transmission from the mother through the placenta just before or during delivery [21], while neonatal malaria, which can occur within the first 28 days of life, is due to an infective mosquito bite after birth [21]. Differentiating between congenital and acquired neonatal malaria can be difficult, especially in areas of intense malaria transmission [22]. Various studies across Africa have demonstrated that 7–10% of newborns may have malaria parasites in their cord blood [8], in some cases either without evidence of an active maternal infection or with parasite genotypes different to those found in the mother. This suggests transplacental passage of parasites followed by clearance from maternal and placental blood, with persistence in the foetus [8,23,24].

Recent reports suggest that malaria in infants under six months of age may not be uncommon, although data on prevalence and outcome are still contradictory [25]. Prevalence of infection can vary between 0% and 27% [9,25-33], while the percentage of deaths attributed to malaria (as determined by verbal autopsy) may be between 20.1% and 46.2% [34]. The occurrence of infection *in utero* is also reflected by the prevalence of splenomegaly at one month of age, which can be as high as 80%, indicating an early development of a splenic response to the infection [5,35].

In Mozambique, clinical malaria incidence in infants aged 1– < 6 months was substantial (320/1,000 child-years at risk in 2003–2004 and 146/1,000 child-years at risk in 2004–2005) [36]. Reports of congenital malaria have predominantly come from Nigeria; the prevalence of parasitaemia in infants in these studies as well as in those from other malaria-endemic regions varied widely – between 0.7% and 46.7% [20,33,37-51]. Parasite densities in neonatal blood were also varied, although most infections were of low density [20,40,42,48-50,52,53]. However, differences in transmission dynamics, small sample sizes and lack of details on quality control and sample selection procedures make the interpretation of these findings difficult [20,25].

Malaria infections (mean geometric parasite density = 533 parasites/μl of blood, as detected by PCR) were common in Ghanaian infants less than 6 months of age (13.6% in newborns; 1.5–9.7% in those aged from 2–26 weeks), although clinical malaria symptoms were rare or uncommon (fever, vomiting, diarrhoea and coughing occurred in 1.8%, 1.8%, 3.0% and 4.8% of infants aged 0–3 months, respectively, and in 4.5%, 3.0%, 8.0% and 10.6% of infants aged 3–6 months) [54]. In Mozambique, although malaria in infants aged under six months represented less than 20% of the total outpatient visits, infants with malaria were admitted in a significantly higher proportion than children aged 1–4 years [36]. The impact of malaria in this age group is also illustrated by a greater difference in mean haematocrit between malaria and non-malaria cases in infants aged from 2–12 months (4.9%) than in children aged 5 years or above (1.8%). Among infants aged under six months attending an urban hospital in Nigeria, 27.1% had a positive blood slide and a significantly lower haematocrit (33.0%) compared with those who were uninfected (35.1%)(p = 0.003). Indeed, a haematocrit of less than 33% was the most common clinical finding among infected infants, while the occurrence of fever was 4% [9].

A systematic review of age-patterns of malaria revealed that as transmission increased, there was a shift of clinical malaria towards younger age groups, regardless of seasonality [55]. As transmission intensity increased and seasonality decreased, severe cases became more frequent in the younger ages. Nevertheless, wherever a comparison between age groups was possible, malaria mortality was higher in younger children, with the peak age shifting towards infants as transmission became more intense [55].

## Clinical signs and symptoms and malaria diagnosis in infants aged under six months

Signs and symptoms of congenital malaria include fever, anaemia, splenomegaly, hepatomegaly, jaundice, vomiting, diarrhoea, poor feeding, restlessness, drowsiness, pallor, respiratory distress, cyanosis, and possibly convulsions [39,56].

Malaria in young infants may be asymptomatic [6,7], or it may be difficult to diagnose because the clinical presentation may mimic other diseases, such as sepsis [33]. Indeed, congenital malaria may be more common in newborns with suspected or confirmed sepsis (28.6%) [38,47]. It is unclear what influence infant HIV infection has on these risks.

In areas with limited resources, the capacity to diagnose malaria in young infants may be limited [57,58], and any issues with quality or accuracy of the diagnostic technique may result in the diagnosis of malaria being missed [59]. In many malaria-endemic countries, infants and children frequently die at home [60] and the cause of death remains undetermined and unrecorded [17,34,61]. Therefore, the overall malaria mortality in infants aged under six months is highly uncertain. As approximately 40% of all child deaths occur in the neonatal period, and many deliveries occur outside health facilities, the proportion of these deaths that is malaria-related is unclear [60].

## Pharmacokinetics of anti-malarials in young infants

Due to the dynamic developmental changes experienced by infants aged under 6 months, the pharmacokinetic profiles of anti-malarials may be different than in older children, and age-dependent dose adjustments may be necessary [62]. For example, gastric emptying is slower in neonates and young infants, and is only comparable with adults after six months of age. Absorption of drugs is also affected by intestinal motor activity and villous formation, both of which mature by week 20 [62]. Drug metabolism may be altered as enzyme systems, such as the hepatic enzymes, are immature (anti-malarials are typically metabolized in the liver) [63], possibly resulting in an enhancement of bioavailability [62]. Activity of such metabolizing enzymes is typically low at birth and rapidly develops over the first 1–2 years of life [64].

## Current national recommendations for treating infants aged under 6 months

As the burden of disease in infants under 6 months of age is not well defined, this age group has been excluded from previous clinical trials and national treatment guidelines on uncomplicated *P*. *falciparum* malaria. Thus, oral anti-malarials recommended as first- and second-line therapies are frequently used off-label, based on the recommended mg/kg dosing schedule for older children [28].

## Issues with currently available anti-malarials

There is little information on the use of artemisinin-based combination therapy (ACT) in young infants, to the extent that many of them carry label restrictions for this age group [63]. Accurate dosing is particularly important, but this is difficult when paediatric formulations are unavailable. Nevertheless, young infants with malaria should receive appropriate treatment. Among the recommended ACT, excluding the combination containing sulphadoxine-pyrimethamine that is not recommended during the first 6 weeks of life [65], there is no evidence of specific serious toxicity [63]. However, additional efforts should be made to establish their safety profile, the correct dosage and formulation, so that young infants with malaria can be managed adequately.

With these issues in mind, it must be considered how ACT could be used to treat malaria in infants aged under six months. There are no official data on how to use ACT in this age group, despite the fact that malaria can occur at a very young age and that ACT offers greater efficacy and tolerability compared with quinine, which is often used in infants with clinical malaria. Furthermore, the use of a unified first-line therapy could offer benefits for healthcare providers with similar anti-malarial use for the whole population, as well as logistical benefits in terms of procurement and distribution. The safety profile of these combination therapies in young infants should be established.

## Preventive treatment

Previous studies have shown that chemoprophylaxis in infants has the potential to reduce malaria-related morbidity and mortality [66]. In The Gambia, infants receiving weekly chloroquine from birth until the age of two years had fewer episodes of malaria, grew better, and had higher haemoglobin levels than the control group [67].

Some studies of intermittent preventive treatment (IPT) in infants (IPTi) have timed the doses of anti-malarial to coincide with routine vaccinations delivered by WHO’s Expanded Programme on Immunization (EPI) at 2, 3 and 9 months. In a pooled analysis of six randomized, placebo-controlled trials of IPTi using sulphadoxine-pyrimethamine delivered at the same time as EPI immunizations, a protective efficacy against clinical malaria of 30.3% was reported [68].

Seasonal malaria chemoprevention (SMC), previously referred to as IPT in children (IPTc), involves the administration of full anti-malarial treatment courses during the malaria season to children aged 3–59 months [69]. The WHO recommends that SMC be used in areas of high seasonal malaria transmission across sub-Saharan Africa, and that treatment with amodiaquine plus sulphadoxine-pyrimethamine should be given at monthly intervals from the start of the transmission season, up to a maximum of four doses [69]. The main potential risks associated with SMC are safety and drug resistance [70]. However, SMC may provide a substantial protective effect against malaria [70], and is a potentially valuable tool that could contribute to reducing the malaria burden in infants aged over three months in areas with seasonal transmission.

The candidate malaria vaccine RTS,S/AS01 administered to infants aged 6–12 weeks at the time of the first vaccination provided modest protection against both uncomplicated and severe malaria [71], casting doubts on whether this vaccine could be included in the routine panel of infants’ immunization [72]. However, additional analyses are needed to understand the reasons of the observed lower efficacy in young infants as compared with older infants and children (aged 5–17 months) [73].

## Future clinical studies

There are several ongoing or planned clinical studies aiming to obtain more accurate estimates of the malaria burden and parasite prevalence in infants aged less than 6 months living under different endemic conditions, as well as the safety and efficacy of currently available ACT in this population. Future longitudinal studies should also characterize infant iron status in relation to subsequent malaria risk, and relate findings to the occurrence of placental malaria.

A multicentre descriptive study is underway, aiming to assess malaria morbidity (prevalence and incidence) and corresponding risk factors in young infants, compared with their siblings, in areas of seasonal low (The Gambia) and high (Guinea) malaria transmission, and in an area with intense, perennial transmission (Benin).

A multicentre trial will evaluate the safety, pharmacokinetics and efficacy of dispersible artemether-lumefantrine tablets in infants weighing under 5 kg with uncomplicated *P*. *falciparum* malaria. This trial, sponsored by Novartis and in association with MMV, commenced in 2012 and involves sites in Benin, Burkina Faso, the Democratic Republic of the Congo, Nigeria and Togo.

Another study planned at the College of Medicine at the University of Malawi will assess the population pharmacokinetics and the safety and efficacy of ACT in infants weighing under 5 kg or aged less than 6 months.

Knowledge gained from such studies will assist in guiding the development of evidence-based treatment guidelines for this population.

## Conclusions

The perception that malaria is uncommon in young infants has resulted in the paucity of information currently available and the lack of evidence-based treatment guidelines in this population. Many children are dying before malaria is diagnosed. In resource-constrained settings, diagnostic techniques may not be available, or malaria may not be recognized due to overlapping symptoms with other neonatal illnesses such as sepsis [33]. Malaria in infants aged under six months is not a rare occurrence in endemic areas and its burden may be underestimated. Therefore, it is necessary to collect reliable data on the malaria burden in this population. Awareness by health professionals should increase so that any infant aged under 6 months brought to a health facility in a malaria-endemic area with unexplained fever or suspected sepsis should be systematically screened for malaria. Current policy for malaria diagnosis dictates that all fevers in all age groups and settings should be tested for malaria before treatment is initiated.

ACT is being used off-label in this vulnerable patient group, with physicians using a pragmatic approach to guide dosing. At this time, a clear recommendation on the use of ACT could be difficult, due to a lack of data in infants with a body weight of less than 5 kg (less than 4.5 kg for artesunate plus amodiaquine). A more accurate estimate of the disease burden and parasite prevalence, risk factors for infection including iron status, as well as the pharmacokinetic profiles, safety and efficacy of currently available ACT in early infancy from data provided by clinical trials that are planned or underway will contribute to the development of evidence-based treatment guidelines for this population, as well as aid the research and development of new drugs. It is definitely worth providing malaria control strategies that are aimed directly at this vulnerable age group, in a bid to reduce infant mortality.

## Abbreviations

ACT: Artemisinin-based combination therapy; EPI: Expanded Programme on Immunization; HbF: Haemoglobin F; HIV: Human Immunodeficiency Virus; IgA: Immunoglobulin A; IgG: Immunoglobulin G; IPT: Intermittent preventive treatment; IPTc: Intermittent preventive treatment in children; IPTi: Intermittent preventive treatment in infants; MMV: Medicines for Malaria Venture; pABA: Para-aminobenzoic acid; PCR: Polymerase Chain Reaction; PfEMP-1: Plasmodium falciparum erythrocyte membrane protein-1; SMC: Seasonal malaria chemoprevention; WHO: World Health Organization.

## Competing interests

UD has been invited as speaker at symposia organized by Sigma Tau and Novartis Pharma, and has received a research grant from Sigma Tau. KH is an employee of Novartis Pharmaceuticals Corporation, East Hanover, NJ, USA. This declaration is made in the interest of full disclosure and not because the author considers this to be a competing interest. The other authors have no competing interest to declare.

## Authors’ contributions

All authors met International Committee of Medical Journal Editors criteria for authorship. All authors contributed to the development of the outline, revised the manuscript critically, and read and approved the final manuscript.

## References

[B1] WHO World malaria report 2011 World Health Organization, Geneva

[B2] Macdonald G The analysis of malaria parasite rates in infants Trop Dis Bull 1950 47 915 938 14798657

[B3] Foll CV Application of malariometric data obtained from longitudinal studies on infants in northern Nigeria Bull World Health Organ 1968 38 255 265 5302301 PMC2554314

[B4] Thomson JG Malaria in nyasaland: (section of tropical diseases and parasitology) Proc R Soc Med 1935 28 391 404 19990138 10.1177/003591573502800424 PMC2205285

[B5] Brabin B An analysis of malaria parasite rates in infants: 40 years after Macdonald Trop Dis Bull 1990 87 1 21 14798657

[B6] Biggar RJ Collins WE Campbell CC The serological response to primary malaria infection in urban Ghanaian infants AmJTrop Med Hyg 1980 29 720 724 10.4269/ajtmh.1980.29.720 7435781

[B7] Sehgal VM Siddjiqui WA Alpers MP A seroepidemiological study to evaluate the role of passive maternal immunity to malaria in infants Trans R Soc Trop Med Hyg 1989 83 Suppl 105 106 2696154 10.1016/0035-9203(89)90616-0

[B8] Fischer PR Malaria and newborns J Trop Pediatr 2003 49 132 134 10.1093/tropej/49.3.132 12848200

[B9] Afolabi BM Salako LA Mafe AG Ovwigho UB Rabiu KA Sanyaolu NO Ibrahim MM Malaria in the first 6 months of life in urban African infants with anemia AmJTrop Med Hyg 2001 65 822 827 10.4269/ajtmh.2001.65.822 11791980

[B10] Riley EM Wagner GE Akanmori BD Koram KA Do maternally acquired antibodies protect infants from malaria infection? Parasite Immunol 2001 23 51 59 10.1046/j.1365-3024.2001.00364.x 11240896

[B11] Riley EM Wagner GE Ofori MF Wheeler JG Akanmori BD Tetteh K McGuinness D Bennett S Nkrumah FK Anders RF Koram KA Lack of association between maternal antibody and protection of African infants from malaria infection Infect Immun 2000 68 5856 5863 10.1128/IAI.68.10.5856-5863.2000 10992495 PMC101547

[B12] Doolan DL Dobano C Baird JK Acquired immunity to malaria Clin Microbiol Rev 2009 22 13 36 Table 10.1128/CMR.00025-08 19136431 PMC2620631

[B13] Kassim OO Ako-Anai KA Torimiro SE Hollowell GP Okoye VC Martin SK Inhibitory factors in breastmilk, maternal and infant sera against in vitro growth of Plasmodium falciparum malaria parasite J Trop Pediatr 2000 46 92 96 10.1093/tropej/46.2.92 10822935

[B14] Gitau GM Eldred JM Malaria in pregnancy: clinical, therapeutic and prophylactic considerations Obstet Gynaecol 2005 7 5 11 10.1576/toag.7.1.005.27036

[B15] Amaratunga C Lopera-Mesa TM Brittain NJ Cholera R Arie T Fujioka H Keefer JR Fairhurst RM A role for fetal hemoglobin and maternal immune IgG in infant resistance to Plasmodium falciparum malaria PLoS One 2011 6 e14798 10.1371/journal.pone.0014798 21532754 PMC3075246

[B16] Gwamaka M Kurtis JD Sorensen BE Holte S Morrison R Mutabingwa TK Fried M Duffy PE Iron deficiency protects against severe Plasmodium falciparum malaria and death in young children Clin Infect Dis 2012 54 1137 1144 10.1093/cid/cis010 22354919 PMC3309886

[B17] Bardaji A Sigauque B Sanz S Maixenchs M Ordi J Aponte JJ Mabunda S Alonso PL Menéndez C Impact of malaria at the end of pregnancy on infant mortality and morbidity J Infect Dis 2011 203 691 699 10.1093/infdis/jiq049 21199881 PMC3071276

[B18] Schwarz NG Adegnika AA Breitling LP Gabor J Agnandji ST Newman RD Lell B Issifou S Yazdanbakhsh M Luty AJ Kremsner PG Grobusch MP Placental malaria increases malaria risk in the first 30 months of life Clin Infect Dis 2008 47 1017 1025 10.1086/591968 18781874

[B19] Malhotra I Dent A Mungai P Wamachi A Ouma JH Narum DL Muchiri E Tisch DJ King CL Can prenatal malaria exposure produce an immune tolerant phenotype? A prospective birth cohort study in Kenya PLoS Med 2009 6 e1000116 10.1371/journal.pmed.1000116 19636353 PMC2707618

[B20] Falade C Mokuolu O Okafor H Orogade A Falade A Adedoyin O Oguonu T Aisha M Hamer DH Callahan MV Epidemiology of congenital malaria in Nigeria: a multi-centre study Trop Med Int Health 2007 12 1279 1287 10.1111/j.1365-3156.2007.01931.x 17956542

[B21] Mukhtar M The growing incidence of neonatal malaria–a situational review in developing countries Niger J Med 2007 16 25 30 17563964

[B22] Fischer PR Nyirjesy P Toko RM Congenital malaria in twins West J Med 1995 163 395 396 7483614 PMC1303154

[B23] Fischer PR Congenital malaria: an African survey Clin Pediatr (Phila) 1997 36 411 413 10.1177/000992289703600706 9241479

[B24] Kamwendo DD Dzinjalamala FK Snounou G Kanjala MC Mhango CG Molyneux ME Rogerson SJ Plasmodium falciparum: PCR detection and genotyping of isolates from peripheral, placental, and cord blood of pregnant Malawian women and their infants Trans R Soc Trop Med Hyg 2002 96 145 149 10.1016/S0035-9203(02)90284-1 12055802

[B25] Mwaniki MK Talbert AW Mturi FN Berkley JA Kager P Marsh K Newton CR Congenital and neonatal malaria in a rural Kenyan district hospital: an eight-year analysis Malar J 2010 9 313 10.1186/1475-2875-9-313 21054891 PMC2988044

[B26] Dicko-Traoré F Syla M Djimde AA Diakité AA Diawara M Togo B Togo P Dara A Dama S Traoré K Traoré S Sissoko S Poudiougo B Sidibé T Keita MM Doumbo O [Congenital and neonatal malaria in sub-sahara Africa, a scarce event?] J Pediatr Pueric 2011 24 57 61

[B27] Klein Klouwenberg PM Oyakhirome S Schwarz NG Glaser B Issifou S Kiessling G Klöpfer A Kremsner PG Längin M Lassmann B Necek M Pötschke M Ritz A Grobusch MP Malaria and asymptomatic parasitaemia in Gabonese infants under the age of 3 months Acta Trop 2005 95 81 85 10.1016/j.actatropica.2005.05.003 15950165

[B28] Larru B Molyneux E ter Kuile FO Taylor T Molyneux M Terlouw DJ Malaria in infants below six months of age: retrospective surveillance of hospital admission records in Blantyre. Malawi Malar J 2009 8 310 10.1186/1475-2875-8-310 20038299 PMC2805692

[B29] Nankabirwa V Tylleskar T Nankunda J Engebretsen IM Sommerfelt H Tumwine JK Malaria parasitaemia among infants and its association with breastfeeding peer counselling and vitamin A supplementation: a secondary analysis of a cluster randomized trial PLoS One 2011 6 e21862 10.1371/journal.pone.0021862 21760916 PMC3131393

[B30] Pedro R Akech S Fegan G Maitland K Changing trends in blood transfusion in children and neonates admitted in Kilifi District Hospital. Kenya Malar J 2010 9 307 10.1186/1475-2875-9-307 21034494 PMC2991344

[B31] Runsewe-Abiodun IT Ogunfowora OB Fetuga BM Neonatal malaria in Nigeria–a 2 year review BMC Pediatr 2006 6 19 10.1186/1471-2431-6-19 16768801 PMC1523330

[B32] van Eijk AM Ayisi JG ter Kuile FO Slutsker L Shi YP Udhayakumar V Otieno JA Kager PA Lal RB Steketee RW Nahlen BL HIV, malaria, and infant anemia as risk factors for postneonatal infant mortality among HIV-seropositive women in Kisumu. Kenya J Infect Dis 2007 196 30 37 10.1086/518441 17538880

[B33] Ojukwu JU Ezeonu CT Ogbu CN Severe malaria in neonates masquerading as septicaemia Nigerian J Paediatr 2004 31 48 55

[B34] Abdullah S Adazu K Masanja H Diallo D Hodgson A Ilboudo-Sanogo E Nhacolo A Owusu-Agyei S Thompson R Smith T Binka FN Patterns of age-specific mortality in children in endemic areas of sub-Saharan Africa AmJTrop Med Hyg 2007 77 99 105 18165480

[B35] Corkill JA Brabin BJ MacGregor DF Alpers MP Milner RD Newborn splenic volumes vary under different malaria endemic conditions Arch Dis Child 1989 64 541 545 10.1136/adc.64.4.541 2665658 PMC1791947

[B36] Guinovart C Bassat Q Sigauque B Aide P Sacarlal J Nhampossa T Bardají A Nhacolo A Macete E Mandomando I Aponte JJ Menéndez C Alonso PL Malaria in rural Mozambique. Part I: children attending the outpatient clinic Malar J 2008 7 36 10.1186/1475-2875-7-36 18302770 PMC2268704

[B37] Adja EA Dick FA N’guessan R [Epidemiological study of the malaria at the neonatal period in the teaching hospital of Yopougon–Republic of Cote d’Ivoire] (in French) Mali Med 2009 24 36 39 20093219

[B38] Ekanem AD Anah MU Udo JJ The prevalence of congenital malaria among neonates with suspected sepsis in Calabar. Nigeria Trop Doct 2008 38 73 76 10.1258/td.2007.005274 18453488

[B39] Ibhanesebhor SE Clinical characteristics of neonatal malaria J Trop Pediatr 1995 41 330 333 10.1093/tropej/41.6.330 8606438

[B40] Lesi FE Mukhtar MY Iroha EU Egri-Okwaji MT Clinical presentation of congenital malaria at the Lagos university teaching hospital Niger J Clin Pract 2010 13 134 138 20499743

[B41] Mosha TCE Ntarukimana D John M Prevalence of congenital malaria among neonates at morogoro regional hospital, morogoro. Tanzania Tanzania J Health Res 2010 12 1 10 10.4314/thrb.v12i4.51792 24409631

[B42] Mukhtar MY Lesi FE Iroha EU Egri-Okwaji MT Mafe AG Congenital malaria among inborn babies at a tertiary centre in Lagos. Nigeria J Trop Pediatr 2006 52 19 23 10.1093/tropej/fmi044 15927946

[B43] Mwangoka GW Kimera SI Mboera LE Congenital Plasmodium falciparum infection in neonates in Muheza District. Tanzania Malar J 2008 7 117 10.1186/1475-2875-7-117 18598342 PMC2474641

[B44] Obiajunwa PO Owa JA Adeodu OO Prevalence of congenital malaria in Ile-ife. Nigeria J Trop Pediatr 2005 51 219 222 10.1093/tropej/fmi003 15980030

[B45] Oduwole OA Ejezie GC Odey FA Oringanje CM Nwakanma D Bello S Oriero E Okebe J Alaribe AA Etuk S Meremikwu M Congenital malaria in Calabar, Nigeria: the molecular perspective AmJTrop Med Hyg 2011 84 386 389 10.4269/ajtmh.2011.10-0253 PMC3042812 21363974

[B46] Okafor UH Oguonu T Onah HE Risk factors associated with congenital malaria in Enugu. South eastern Nigeria J Obstet Gynaecol 2006 26 612 616 10.1080/09638280600902893 17071423

[B47] Okechukwu AA Olateju EK Olutunde EO Congenital malaria among newborns admitted for suspected neonatal sepsis in Abuja Nigerian J Paediatr 2011 38 82 89

[B48] Omalu IC Mgbemena C Mgbemena A Ayanwale V Olayemi IK Lateef A Chukwuemeka VI Prevalence of congenital malaria in Minna, north central Nigeria J Trop Med 2012 201 2 274142 21876706 10.1155/2012/274142 PMC3157756

[B49] Orogade AA Falade CO Okafor HU Mokuolu OA Mamman AI Ogbonu TA Ogunkunle OO Ernest KS Callahan MV Hamer DH Clinical and laboratory features of congenital malaria in Nigeria J Pediatr Infect Dis 2008 3 181 187

[B50] Pineros-Jimenez JG Alvarez G Tobon A Arboleda M Carrero S Blair S Congenital malaria in Uraba. Colombia Malar J 2011 10 239 10.1186/1475-2875-10-239 21846373 PMC3177814

[B51] Sule-Odu AO Ogunledun A Olatunji AO Impact of asymptomatic maternal malaria parasitaemia at parturition on perinatal outcome J Obstet Gynaecol 2002 22 25 28 10.1080/01443610120101664 12521723

[B52] Larkin GL Thuma PE Congenital malaria in a hyperendemic area AmJTrop Med Hyg 1991 45 587 592 10.4269/ajtmh.1991.45.587 1951868

[B53] Akindele JA Sowunmi A Abohweyere AE Congenital malaria in a hyperendemic area: a preliminary study Ann Trop Paediatr 1993 13 273 276 7505553 10.1080/02724936.1993.11747658

[B54] Wagner G Koram K McGuinness D Bennett S Nkrumah F Riley E High incidence of asymptomatic malara infections in a birth cohort of children less than one year of age in Ghana, detected by multicopy gene polymerase chain reaction AmJTrop Med Hyg 1998 59 115 123 10.4269/ajtmh.1998.59.115 9684638

[B55] Carneiro I Roca-Feltrer A Griffin JT Smith L Tanner M Schellenberg JA Greenwood B Schellenberg D Age-patterns of malaria vary with severity, transmission intensity and seasonality in sub-Saharan Africa: a systematic review and pooled analysis PLoS One 2010 5 e8988 10.1371/journal.pone.0008988 20126547 PMC2813874

[B56] Hashemzadeh A Heydarian F Congenital malaria in a neonate Arch Iranian Med 2005 8 226 228

[B57] Hailegiorgis B Girma S Melaku Z Teshi T Demeke L Gebresellasie S Yadeta D Tibesso G Whitehurst N Yamo E Carter J Reithinger R Laboratory malaria diagnostic capacity in health facilities in five administrative zones of oromia regional state. Ethiopia Trop Med Int Health 2010 15 1449 1457 10.1111/j.1365-3156.2010.02646.x 21040254

[B58] Ishengoma DR Derua YA Rwegoshora RT Tenu F Massaga JJ Mboera LE Magesa SM The performance of health laboratories and the quality of malaria diagnosis in six districts of Tanzania Ann Trop Med Parasitol 2010 104 123 135 10.1179/136485910X12607012373993 20406579

[B59] Uneke CJ Congenital Plasmodium falciparum malaria in sub-Saharan Africa: a rarity or frequent occurrence? Parasitol Res 2007 101 835 842 10.1007/s00436-007-0577-9 17549517

[B60] Lawn JE Cousens S Zupan J 4 million neonatal deaths: when? Where? Why? Lancet 2005 365 891 900 10.1016/S0140-6736(05)71048-5 15752534

[B61] Sacarlal J Nhacolo AQ Sigauque B Nhalungo DA Abacassamo F Sacoor CN Aide P Machevo S Nhampossa T Macete EV Bassat Q David C Bardají A Letang E Saúte F Aponte JJ Thompson R Alonso PL A 10 year study of the cause of death in children under 15 years in Manhica. Mozambique BMC Publ Health 2009 9 67 10.1186/1471-2458-9-67 PMC2656537 19236726

[B62] Kearns GL Abdel-Rahman SM Alander SW Blowey DL Leeder JS Kauffman RE Developmental pharmacology–drug disposition, action, and therapy in infants and children N Engl J Med 2003 349 1157 1167 10.1056/NEJMra035092 13679531

[B63] WHO Guidelines for the treatment of Malaria 2010 Second World Health Organization, Geneva, Switzerland

[B64] Milsap RL Jusko WJ Pharmacokinetics in the infant Environ Health Perspect 1994 102 Suppl 11 107 110 10.1289/ehp.94102s11107 7737034 PMC1566769

[B65] Menendez C Mayor A Congenital malaria: the least known consequence of malaria in pregnancy Semin Fetal Neonatal Med 2007 12 207 213 10.1016/j.siny.2007.01.018 17483042

[B66] Geerligs PD Brabin BJ Eggelte TA Analysis of the effects of malaria chemoprophylaxis in children on haematological responses, morbidity and mortality Bull World Health Organ 2003 81 205 216 12764517 PMC2572421

[B67] McGregor IA Gilles HM Walters JH Davies AH Pearson FA Effects of heavy and repeated malarial infections on Gambian infants and children; effects of erythrocytic parasitization BMJ 1956 2 686 692 10.1136/bmj.2.4994.686 13356045 PMC2035220

[B68] Aponte JJ Schellenberg D Egan A Breckenridge A Carneiro I Critchley J Danquah I Dodoo A Kobbe R Lell B May J Premji Z Sanz S Sevene E Soulaymani-Becheikh R Winstanley P Adjei S Anemana S Chandramohan D Issifou S Mockenhaupt F Owusu-Agyei S Greenwood B Grobusch MP Kremsner PG Macete E Mshinda H Newman RD Slutsker L Tanner M Alonso P Menendez C Efficacy and safety of intermittent preventive treatment with sulfadoxine-pyrimethamine for malaria in African infants: a pooled analysis of six randomised, placebo-controlled trials Lancet 2009 374 1533 1542 10.1016/S0140-6736(09)61258-7 19765816

[B69] WHO Global Malaria Programme WHO Policy Recommendation: Seasonal Malaria Chemoprevention (SMC) for Plasmodium falciparum malaria control in highly seasonal transmission areas of the Sahel sub-region in Africa 2012 http://www.who.int/malaria/publications/atoz/smc_policy_recommendation_en_032012.pdf

[B70] Gosling RD Okell L Mosha J Chandramohan D The role of antimalarial treatment in the elimination of malaria Clin Microbiol Infect 2011 17 1617 1623 10.1111/j.1469-0691.2011.03660.x 21951597

[B71] The RTS,S Clinical Trials Partnership A phase 3 trial of RTS,S/AS01 malaria vaccine in African infants N Engl J Med 2012 10.1056/NEJMoa1208394 PMC10915853 23136909

[B72] Daily JP Malaria vaccine trials - beyond efficacy end points N Engl J Med 2012 10.1056/NEJMe1213392 23136910

[B73] Agnandji ST Lell B Soulanoudjingar SS Fernandes JF Abossolo BP Conzelmann C Methogo BG Doucka Y Flamen A Mordmüller B Issifou S Kremsner PG Sacarlal J Aide P Lanaspa M Aponte JJ Nhamuave A Quelhas D Bassat Q Mandjate S Macete E Alonso P Abdulla S Salim N Juma O Shomari M Shubis K Machera F Hamad AS Minja R Mtoro A Sykes A Ahmed S Urassa AM Ali AM Mwangoka G Tanner M Tinto H D’Alessandro U Sorgho H Valea I Tahita MC Kaboré W Ouédraogo S Sandrine Y Guiguemdé RT Ouédraogo JB Hamel MJ Kariuki S Odero C Oneko M Otieno K Awino N Omoto J Williamson J Muturi-Kioi V Laserson KF Slutsker L Otieno W Otieno L Nekoye O Gondi S Otieno A Ogutu B Wasuna R Owira V Jones D Onyango AA Njuguna P Chilengi R Akoo P Kerubo C Gitaka J Maingi C Lang T Olotu A Tsofa B Bejon P Peshu N Marsh K Owusu-Agyei S Asante KP Osei-Kwakye K Boahen O Ayamba S Kayan K Owusu-Ofori R Dosoo D Asante I Adjei G Adjei G Chandramohan D Greenwood B Lusingu J Gesase S Malabeja A Abdul O Kilavo H Mahende C Liheluka E Lemnge M Theander T Drakeley C Ansong D Agbenyega T Adjei S Boateng HO Rettig T Bawa J Sylverken J Sambian D Agyekum A Owusu L Martinson F Hoffman I Mvalo T Kamthunzi P Nkomo R Msika A Jumbe A Chome N Nyakuipa D Chintedza J Ballou WR Bruls M Cohen J Guerra Y Jongert E Lapierre D Leach A Lievens M Ofori-Anyinam O Vekemans J Carter T Leboulleux D Loucq C Radford A Savarese B Schellenberg D Sillman M Vansadia P RTS,S Clinical Trials Partnership: First results of phase 3 trial of RTS,S/AS01 malaria vaccine in African children N Engl J Med 2011 365 1863 1875 22007715 10.1056/NEJMoa1102287

